# Bi-compartmental evaluation of bacterial prints in infectious diseases – study hypothesis

**DOI:** 10.1186/1471-2334-14-S7-P10

**Published:** 2014-10-15

**Authors:** Oana Săndulescu, Dragoş Florea, Anca Streinu-Cercel, Dan Oțelea, Alina Cristina Neguț, Petre Iacob Calistru, Adrian Streinu-Cercel

**Affiliations:** 1 Carol Davila University of Medicine and Pharmacy, Bucharest, Romania; 2 National Institute for Infectious Diseases "Prof. Dr. Matei Balş", Bucharest, Romania

## Background

PCR-based tools for detection of bacterial infections can considerably shorten the timespan from patient admission to initiation of targeted antibiotherapy. As culture-based methods are operator-sensitive and yield lower rates of identification in longer timespans, dependent on species-specific growth rates, there is a need for new techniques leading to rapid identification of the pathogenic agent and its antimicrobial susceptibility profile.

PLEX-ID (Abbott Molecular Inc, Des Plaines, USA) ensures bacterial identification in a matter of hours, directly from clinical specimens, through PCR amplification and electrospray ionization-mass spectrometry.

## Study hypothesis

Chronic bacterial infections have long been considered an important issue in clinical practice, particularly when foreign bodies promote biofilm formation. However, other mechanisms of bacterial latency such as phenotypic variants leading to persister cells have also been described [1], and are increasingly encountered in the clinic. Small colony variants or persister cells with low metabolic activity can associate significant delays in laboratory identification through culture-based techniques. Therefore, there is a stringent need for the implementation of novel identification methods.

We aim to perform a prospective study on bi-compartmental evaluation of bacterial prints in patients with infectious diseases, within the full range of Carmeli scores [2]. Serial samples will be collected from normally-sterile bodily compartments, at three different time-points: baseline (initiation of antimicrobial therapy), end-of-treatment, 3-week follow-up. PLEX-ID will be performed directly from the samples drawn from at least two normally-sterile bodily compartments, depending on the primary site of infection. Where two normally-sterile or tissue samples are not available, blood samples will be used as control. The decline of bacterial load will be dynamically assessed through semi-quantitative methods and results will be interpreted based on clinical and paraclinical data on patient evolution, and the degree of adequacy between the identified antimicrobial sensitivity profile and the type of therapy administered in clinical practice.

## Expected results

We aim to define particular timelines of bacterial internalization or immune clearance and to pinpoint a critical interval when progression to persistent infection can occur, and when a clinical intervention would be able to stop and avert the chronicisation of bacterial infection.
